# Supplementary material for: Principles of gamma synchrony predict figure–ground perception in texture stimuli
Source: eLife. 2026 Apr 10;14:RP105482. doi: 10.7554/eLife.105482 (PMC13068434; doi:10.7554/eLife.105482)
Supplement: Supplementary file 1. — Detection probability refers to the proportion of simulated datasets in which the posterior probability of an effect exceeded 0.95 in the predicted direction. Type-S error indicates the probability of detecting an effect, but in the wrong direction (sign reversed). Type-M error refers to the ratio of estimated to true effect size when detected. A value of 1 indicates no deviation, whereas values larger (smaller) than 1 indicate that effects are over (under) estimated. [file elife-105482-supp1.docx]

**Supplementary File** 1**: Design analysis of main analysis in session 1.** Detection probability refers to the proportion of simulated datasets in which the posterior probability of an effect exceeded 0.95 in the predicted direction. Type-S error indicates the probability of detecting an effect, but in the wrong direction (sign reversed). Type-M error refers to the ratio of estimated to true effect size when detected. A value of 1 indicates no deviation whereas values larger (smaller) than 1 indicate that effects are over (under) estimated.

| **Sample size** | **Detection probability** | | | **Type-S error** | | | **Type-M error** | | |
| --- | --- | --- | --- | --- | --- | --- | --- | --- | --- |
|  | **Contrast Heterogeneity** | **Grid Coarseness** | **Interaction** | **Contrast Heterogeneity** | **Grid Coarseness** | **Interaction** | **Contrast Heterogeneity** | **Grid Coarseness** | **Interaction** |
| 4 | 0.74 | 0.54 | 0.48 | 0.00 | 0.00 | 0.00 | 0.94 | 1.10 | 1.11 |
| 6 | 0.92 | 0.82 | 0.84 | 0.00 | 0.00 | 0.00 | 1.11 | 1.01 | 1.03 |
| 8 | 0.96 | 0.92 | 0.92 | 0.00 | 0.00 | 0.00 | 0.99 | 0.96 | 1.02 |
